# The initial pharmaceutical development of an artesunate/amodiaquine oral formulation for the treatment of malaria: a public-private partnership

**DOI:** 10.1186/1475-2875-10-142

**Published:** 2011-05-23

**Authors:** Catherine Lacaze, Tina Kauss, Jean-René Kiechel, Antonella Caminiti, Fawaz Fawaz, Laurent Terrassin, Sylvie Cuart, Luc Grislain, Visweswaran Navaratnam, Bellabes Ghezzoul, Karen Gaudin, Nick J White, Piero L Olliaro, Pascal Millet

**Affiliations:** 1 Ellipse Pharmaceuticals, Pessac, France; 2 EA Pharmaceutical and Analytical Development Applied to Neglected Diseases and Counterfeit drugs, University Bordeaux Segalen, Bordeaux, France; 3 Drugs for Neglected Diseases initiative (DNDi), Geneva, Switzerland; 4 Tropival, University Bordeaux Segalen, Bordeaux, France; 5 Centre for Drug Research, University Sains Malaysia, Malaysia; 6 Pharmacie, CHU Pellegrin, Bordeaux, France; 7 Centre for Tropical Medicine and Vaccinology, Nuffield Department of Medicine, University of Oxford, Churchill Hospital, Oxford, UK; 8 UNICEF/UNDP/WB/WHO Special Program for Research & Training in Tropical Diseases (TDR), Geneva, Switzerland

## Abstract

**Background:**

Artemisinin-based combination therapy is currently recommended worldwide for the treatment of uncomplicated malaria. Fixed-dose combinations are preferred as they favour compliance. This paper reports on the initial phases of the pharmaceutical development of an artesunate-amodiaquine (ASAQ) bilayer co-formulation tablet, undertaken following pre-formulation studies by a network of scientists and industrials from institutions of both industrialized and low income countries.

**Methods:**

Pharmaceutical development was performed by a research laboratory at the University Bordeaux Segalen, School of Pharmacy, for feasibility and early stability studies of various drug formulations, further transferred to a company specialized in pharmaceutical development, and then provided to another company for clinical batch manufacturing. The work was conducted by a regional public-private not-for-profit network (TropiVal) within a larger Public Private partnership (the FACT project), set up by WHO/TDR, Médecins Sans Frontières and the Drugs for Neglected Disease initiative (DND*i*).

**Results:**

The main pharmaceutical goal was to combine in a solid oral form two incompatible active principles while preventing artesunate degradation under tropical conditions. Several options were attempted and failed to provide satisfactory stability results: incorporating artesunate in the external phase of the tablets, adding a pH regulator, alcoholic wet granulation, dry granulation, addition of an hydrophobic agent, tablet manufacturing in controlled conditions. However, long-term stability could be achieved, in experimental batches under GMP conditions, by physical separation of artesunate and amodiaquine in a bilayer co-formulation tablet in alu-alu blisters. Conduction of the workplan was monitored by DND*i*.

**Conclusions:**

Collaborations between research and industrial groups greatly accelerated the process of development of the bi-layered ASAQ tablet. Lack of public funding was the main obstacle hampering the development process, and no intellectual property right was claimed. This approach resulted in a rapid technology transfer to the drug company Sanofi-Aventis, finalizing the process of development, registration and WHO pre-qualification of the fixed-dose co-formulation together with DND*i*. The bi-layered tablet is made available under the names of Coarsucam^® ^and Artesunate amodiaquine Winthrop^®^, Sanofi-Aventis. The issue related to the difficulty of public institutions to valorise their participation in such initiative by lack of priority and funding of applied research is discussed.

## Background

The World Health Organization (WHO) currently recommends artemisinin-based combination therapy (ACT) for the treatment of uncomplicated *Plasmodium falciparum *malaria [1]. Virtually all malaria endemic countries have adopted by now an ACT as first-line treatment. Initially only individually formulated compounds were available; subsequently, products were co-blistered. In both cases patient's adherence may be compromised. This called for the development of fixed-dose co-formulations (FDC). However combining two anti-malarial drugs into a single tablet may be challenging, and requires compliance to stringent regulatory requirements.

The first FDC ACT ever registered was artemether plus lumefantrine (Coartem^® ^and Riamet^®^, Novartis) launched in 2001. Other widely-used combinations at that time were artesunate plus amodiaquine and artesunate plus mefloquine.

The Special Programme for Research and Training in Tropical Diseases of the WHO (TDR) initiated exploratory pharmaceutical work on anti-malarial drug associations based on AS and AQ [2], under its Resistance And Policy (RAP) taskforce at the University of Bordeaux 2, France local consortium TropiVal [3] and the University Sains Malaysia.

**Figure 1:**
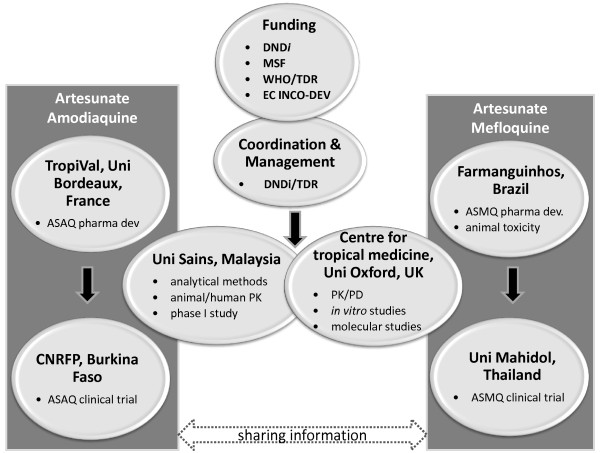
**FACT Partnerships**. Key: Uni = University; pharma dev = pharmaceutical development; PK = pharmacokinetics, PD = pharmacodynamics.

Later, this project became part of the fixed artemisinin-based combination treatment (FACT) project, led by the DND*i *and TDR. The FACT project received initial funding of the European Commission (INCO-DEV) and MSF, and then became largely DND*i *self-funded. The FACT aimed at developing two fixed-dose combinations: artesunate plus amodiaquine (ASAQ) and artesunate plus mefloquine (ASMQ) [4,5]. Initially, the network involved only partners from the public and non-for-profit sector (Figure 1).

The target product profile (see Table 1) set for the ASAQ was for a fixed-dose fast-release oral formulation combining AS and AQ for paediatric use in tropical conditions (climatic zone IV, hot and humid; [6]). Beside stability, the target product profile requires also adapted dose ranges for the tablets for both active components, minimal excipient quantities to keep tablets small as well as rapid disintegration for ease of use particularly in children. This paper relates the development of an oral ASAQ formulation. The local consortium in Bordeaux TropiVal [3] comprised the Laboratory of Biopharmacy, Bordeaux 2 University, School of Pharmacy, France, and later ELLIPSE Pharmaceuticals, a university start-up specialized in drug development and process validation. Analytical methods were developed by the University Sains Malaysia (USM) and then transferred to Bordeaux2 University. The University of Oxford, UK and the National Centre for Research and Training in Malaria (CNRFP), Burkina-Faso, participated in subsequent phases of development. Tablet strengths were designed to fit predictions based on optimal dose ranges and anthropometric (age/weight) parameters [7]. Early investigations on the development of a fixed co-formulation focused on the compatibility between artesunate and amodiaquine, and feasibility and stability studies of a fixed-dose co-formulation [2].

**Table 1:** Target product profile of developed ASAQ formulation

Attribute	Target product characteristics
API	AS and AQ

Population targeted	pediatric and adult

Posology	based on [7] ASAQ low strength 25/67.5 mg (equivalent AQ base) for children ASAQ high strength 100/270 mg (equivalent AQ base) for older children and adults

Drug release	rapid disintegration and fast drug release (> 75% in 45 minutes in vitro dissolution test, Pharmacopoeia standards)

Drug form	compatible with pediatric use: minimal size for facilitating swallowing; a fixed dose combination

Stability/Conditioning	compatible with climatic zone IV (tropical conditions): ICH Q1A(R2)

Cost	< 1$

This paper recounts the development process that led to the formulation of ASAQ in bilayer tablets up to the clinical batches used for initial clinical studies performed in Burkina Faso [8], and subsequently transferred to commercial partners identified by DND*i *(Sanofi-Aventis in 2005, Bompart F, Kiechel JR, Sebbag R, Pecoul B: Innovative public-private partnership to maximize the delivery of anti-malarial medicines: lessons learned from the ASAQ Winthrop experience, submitted).

## Methods

### Chemicals

Artesunate (AS) was purchased from Knoll, Switzerland (currently Abbott Laboratories) and amodiaquine hydrochloride (AQH) salt (AQ.2HCl.2H_2_O) and amodiaquine base (AQb) were obtained from Parke Davis, Senegal and Ipca, India. Dihydroartemisinin (DHA), AS main metabolite, was provided by Knoll. Solvents and buffers were of analytical grade. Tablet excipients were of pharmaceutical grade. The physicochemical properties of AS and AQ were characterized and reported previously [2].

### Analytical methods

#### Artesunate and DHA assay

AS was determined by HPLC with UV detection on Varian ProStar model chromatograph apparatus equipped with Hypersil C4 column, 5 μm, 250 mm × 4.6 mm. Sodium acetate trihydrate 0.05 M/NaOH for pH 5,2/acetonitrile mixture (63/37%V/V) was used as the mobile phase with flow rate of 1.5 ml.min^-1^. The injection volume was 100 μl and the oven temperature 27°C, UV detector was set to 211 nm. In these conditions, the retention time of AS was 8 minutes, that of DHA of 11 and 16 minutes (the α and β DHA epimers, respectively) and that of artemisinin of 10 minutes. The analysis time was 15 minutes.

AS standard solution (1 mg mL^-1 ^in mobile phase) was diluted in mobile phase to obtain calibration curve (n≥5) for the desired concentration range. This method was validated according to ICH guidelines. Further internal specifications were implemented: RSD of 6 consecutive injections < 2%.

#### Amodiaquine assay

AQ was dosed by spectrophotometry using PERKIN ELMER lambda 20 Spectrophotometer at 342 nm with cell thickness of 1 mm. AQ standard solution (89 mg of amodiaquine hydrochloride completed to 50 mL with 1% HCl) was further diluted ten-fold with a 1% hydrochloric acid solution.

### ASAQ tablet preparation and evaluation

#### Development strategy

From pre-formulation studies [2] it was clear that some key parameters, namely water or humidity presence, HCl release or AS AQ contact, have to be controlled for suitable stability of obtained tablets. The development was a series of pharmacotechnical manufacturing processes beginning from the simplest one and going towards more complicated ones to overcome the problems raised regarding target product profile. The aim was to obtain a stable ASAQ tablet with the simplest possible technology. First, direct compression was discarded, then granulation step was considered (aqueous, alcoholic and dry), with or without excipients to enhance tablet stability (pH regulator, hydrophobic agent...). The optimized process ended up with the choice of a bilayer ASAQ tablet.

#### Tablet preparation

In all monolayer ASAQ tablets, sodium croscarmellose (Acdisol^®^) was used as diluent/desintegrant, polyvinylpyrrolidone (PVP K30^® ^or K25^® ^12%/16% aqueous solution) as a binder, colloidal silica (Aerosil^® ^300) as flowing agent, and magnesium stearate was used as lubricant. The different components were sieved through a 1 mm grate, AQ was sieved through a 250 μm mesh diameter siever). All monolayer tablets were compressed using a rotary SVIAC PR6 tablet press equipped with 2 round 13 mm diameter punches.

Several preparation techniques were used to optimize ASAQ tablets. In tablets prepared by wet granulation, internal phase constituents (AS, AQ and a part of Acdisol^® ^for batch n° 20399, AQ and Acdisol^® ^for batch n° 20454) were mixed in a planetary mixer and moistened with 12% PVP K30^® ^solution and 16%PVPK25^® ^solution respectively. The moist mass was then passed through 1.6 mm mesh of oscillating granulator and dried in fluid air bed dryer at 60°C for 35 minutes. After drying, granulate was screened again (0.80 mm mesh), mixed with external phase constituents (lubricants and 1%w/w of Acdisol^® ^for batch 20399, AS and CaCO_3 _in addition in batch n° 20454) before compression.

Dry granulation tablets (batches 30411-30439) were prepared using POWTEC RC 100 × 30 compactor equipped with a rotative calibrator for AQ alone compaction. The plates obtained were calibrated through a 0.8 mm screen and then mixed in planetary mixer with other excipients.

**Figure 2:**
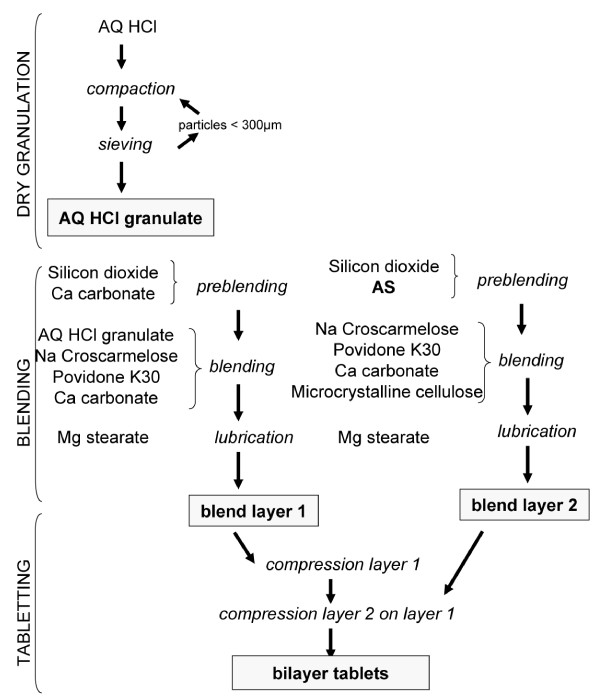
**Bilayer tablets manufacturing process**.

Some batches (30412, 30437, 30439) contained additional colloidal silica (Aerosil^® ^300) as hydrophobic agent and some were manufactured under controlled conditions (batches 30436-30439). The bilayer tablet manufacturing process is summarized in Figure 2. All tablet pharmacotechnical controls (mass uniformity, disintegration time, hardness) were performed according to Pharmacopoeia standards unless specified otherwise.

#### Tablet stability and pre-stability studies

For short-term pre-stability studies, tablets were placed in sealed glass vials at 25°C/60% RH and 40°C/75% RH according to ICH guidelines ICH QA1(R2). In the absence of tropical zone ambient storage department, 60 month stability study of bilayer tablets was carried out in CHU Pellegrin hospital official drug storage department, France at 15 to 25°C and ambient humidity. Tablets were packaged in Alu/Alu blisters for optimized humidity protection. Several conditioned aliquots of each tablet batch were analysed for AS, AQ and DHA content at defined times. Regulatory stability studies for ASAQ formulations were further performed by Sanofi-Aventis.

#### Tablet AS and DHA content determination

10 or 20 tablets were finely powdered in a mortar. An aliquot of 1,4 g was put into a 100 ml volumetric flask, completed after stirring with water/ethanol (40/60%V/V) and filtered through a 0.45 μm filter. 50 μl were injected according to the HPLC AS method described above. AS was quantified by comparison with a test solution of 2 mg/ml in water/ethanol (40/60%V/V). The specifications used were AS content (100 mg or 25 mg) ± 5%w/w (according to ICH specifications) and further internal specifications for DHA content < 3% after 36 months aging (< 1% DHA in short term studies) were applied, as DHA indicated an ongoing AS degradation process.

#### Tablet AQ content determination

10 or 20 tablets were finely powdered in a mortar. An aliquot of 700 mg was put into a 200 ml volumetric flask, dissolved in 150 ml 0.1N HCl sonicated for 10 minutes and electromagnetically stirred for 20 minutes and completed. The obtained solution was filtered through a 0.45 μm filter, diluted ten-fold in HCl 0.1N and analysed using AQ spectrophotometric method described. AQ was quantified comparing to test solution of 88 mg AQH dissolved in 50 ml 0,1N HCl and diluted ten-fold. The specifications were AQ content (270 mg or 67 mg) ± 5% w/w (ICH stability study specifications).

#### ASAQ tablet dissolution test

Dissolution studies were carried out in 500 ml and 900 ml of 11.4 g.l^-1 ^sodium acetate and 0.886 g.l^-1 ^acetic acid aqueous solution adjusted to pH 5.5 for low dosage (25 mg AS 67.5 mg AQ) and high dosage (100 mg AS 270 mg AQ) ASAQ tablets respectively, using European pharmacopoeia apparatus 2 (100 rpm, 37°C). At defined times (15, 30, 45, 60, 90 minutes) samples were withdrawn with 10 μm porous prefilter equipped syringes. Drug concentration in sample was determined using previously described HPLC method for AS and DHA and UV spectrophotometric method for AQ and comparing dissolution samples to standard solutions. The specifications were AQ and AS drug release > 75% at 45 minutes.

## Results

### Direct compression

A direct compression process proved not feasible as AQ compressibility was poor and the high-strength tablets required too large a quantity of excipients for an acceptable tablet size.

### Aqueous wet granulation

The initial wet granulation monolayer tablet formulation containing sodium croscarmellose, PVP K30, magnesium stearate and silicone dioxide was found unstable leading to 9% AS loss after 3 months at 40°C/75%RH [2]. When this process was adapted to industrial scale (modification of wet granulation parameters, flowability and compressibility improvement), AS degradation increased and only 83.7 and 79.6% of AS were found for the high and low-strength ASAQ tablets respectively after 2 months at 40°C/75%RH.

Several approaches were considered to avoid previously identified factors that affect AS degradation (such as humidity, released HCl and AQ contact, [2]): incorporation of AS in the tablet external phase, inclusion of pH regulator, alcoholic wet granulation, dry granulation, increased hydrophobic agents, tablet manufacturing in controlled conditions, physical separation between AS and AQ (bilayer tablets).

### AS in external phase

AS was incorporated in the external tablet phase (after the granulation step) in order to avoid direct contact with water during aqueous wet granulation of AQ. Tablets were found stable during two months at 40°C/75%RH, but 17% AS degradation occurred during the third month.

### pH regulator

**Figure 3:**
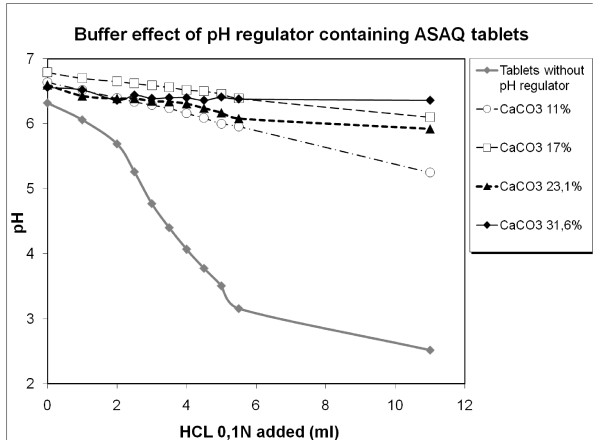
**Buffer effect of ASAQ tablets containing pH regulator (CaCO_3_) while adding HCl 0,1N**.

**Figure 4:**
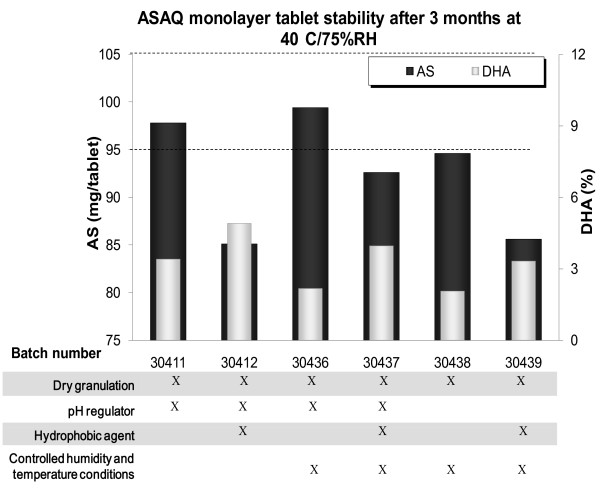
**Tablet AS and DHA content of ASAQ monolayer tablets, after 3 months at 40°C/75%RH blistered**. Key: AS = artesunate; DHA = dihydroartesunate; black dotted lines: specifications of AS content (100 ± 5 mg/tablet). Mean of 10. AS content in black, on the left Y axis; DHA content in grey, on the right Y axis Tablet formulations are summarized in the table under histogrammes.

CaCO_3 _and NaHCO_3 _were tested as pH regulators in order to neutralize the effects of the possible release of HCl which was shown to induce rapid AS degradation [2]. As NaHCO_3 _precipitated during the dissolution test, CaCO_3 _was selected. Increasing quantities of carbonate were studied for the buffer effect conferred to ASAQ tablets while adding HCl in the dissolution medium (Figure 3). 17%w/w was selected as optimal CaCO_3 _tablet content for further investigation combining buffering power and technical ease. When this formulation was submitted to accelerated stability conditions (40°C/75%RH,) AS degradation was delayed compared to tablets without CaCO_3_, but occurred nevertheless after three months. The presence of CaCO_3_, while further improving tablet stability in other dry granulation formulations (see Figure 4, batch n° 30436 with pH regulator vs. 30438 without), was not sufficient to guarantee the absence of AS degradation in the tested conditions.

### Alcoholic wet granulation

To avoid direct water contact, alcoholic granulation was tried. Several formulations were tested and the performances of some of them are summarized in Table 2. Globally, besides avoiding direct contact with water, alcoholic compared to aqueous granulation improved tablets disintegration, which was one of main pharmacotechnical specifications, but increased their friability. No immediate incompatibility was noticed. However, scale transposition of alcoholic granulated tablets was considered to have too many constraints at industrial scale. Stability studies of these formulations were hence not pursued.

**Table 2:** Pharmacotechnical characteristics of ASAQ alcoholic/aqueous granulation tablet formulations with or without binder*

Batch n°	11128G	11213(1)	11129	11128P	11124	11130
Tablet dosage AS/AQ (mg/mg)	100/270	100/270	100/270	25/67,5	25/67,5	25/67,5

Granulation	Aqueous + PVP*	Alcoholic + PVP*	Alcoholic without PVP*	Aqueous + PVP*	Alcoholic + PVP*	Alcoholic without PVP*

Mean tablet weight (mg)	494	480	476	129	123	120

Hardness (N)	73	65	69	72	60	59

Disintegration time (min)	4	< 1	< 1	5	< 3	< 3

Friability (%)	1.25	ND	2.6	0.7	ND	1.04

Drugs released after 45 min of dissolution test (AQ%/AS%)	92/76	94/83	98/91	100/86	ND	100/106

Key: ND = not determined; binder = PVP K30 10% solution

### Dry granulation process

Beyond avoiding water contact, which was the first objective of dry granulation studies, this process was particularly suitable for developed formulation, as a small tablet size was required. AS was added in the external phase of tablets and 17% pH regulator content included. Dry granulation tablet stability was largely improved (> 95% AS and 100% AQ after three months at 40°C/75%RH; batch n° 30411, Figure 4) compared to wet granulation tablets (84% AS; batch n° 20454 Figure 3) with the same formulation (containing pH regulator). Nevertheless 3.4% of DHA found in dry granulation tablets indicated ongoing AS degradation.

### Hydrophobic agent

To further reduce negative water impact on AS stability in ASAQ tablets, higher silicon dioxide percentage (1.9%w/w, contrarily to 0.9%w/w used as flowing agent in initial and all further formulations) was added to AS in external phase to improve its hydrophobic properties. However AS degradation increased in the presence of higher content of silicon dioxide in all tested formulations (e.g. batch n° 30412 *vs*. 30411) (Figure 4).

### Controlled temperature and humidity conditions

To prevent ASAQ tablets from humidity contact, tablets (batch n° 30435 to 30438) were manufactured in controlled conditions (21°C ± 3°C and 32 ± 4%RH). These conditions enhanced AS stability (Figure 4) as AS content was stable after a three-month stability study, and as the DHA content decreased compared to non-controlled conditions (2.2% vs. 3.4% DHA respectively). However, this was considered not enough to warrant further development. Since all the approaches above had failed and since basic quinoline structures (like AQ) as well as the presence of HCl appeared to be involved in AS degradation [2], the physical separation between the two APIs was the next option to be considered.

### ASAQ bilayer tablets

A bilayer ASAQ tablet formulation was developed to minimize the direct contact between AS and AQ. Table 3 summarizes the characteristics of this formulation for both low- and high-strength bilayer ASAQ tablets. The pharmacotechnical properties of the tested formulations are summarized in Table 4.

**Table 3:** Bilayer ASAQ tablet formulation for high dosage and low dosage ASAQ tablets

*Composition*	*Content* *(%w/w)*	*Quantity (mg/tablet)*	
		
***Layer 1 (blend no 30154C01 )***		***high dosage tablet (batch 30154C03)***	***low dosage tablet (batch 30154C04)***
Dry granulated AQH	50.38	352.64	88.16

Sodium Croscarmellose (AcDiSol)	1.40	9.80	2.45

Polyvinylpyrrolidone (PVP K30)	1.14	8.00	2.00

Magnesium stearate	1.32	9.26	2.315

Silicon dioxide (Aerosil 300)	0.11	0.75	0.1875

Ca carbonate	7.71	54.00	13.50

***Total layer 1***	***62.06***	***434.45***	***108.61***

***Layer 2 (blend 30154C03)***			

AS	14.29	100.00	25.00

Polyvinylpyrrolidone (PVP K30)	1.14	8.00	2.00

Sodium Croscarmellose (AcDiSol)	1.40	9.80	2.45

Magnesium stearate	0.43	3.00	0.75

Silicon dioxide (Aerosil 300)	0.11	0.75	0.1875

Calcium carbonate	7.71	54.00	13.50

Microcrystalline cellulose	12.86	90.00	22.50

***Total layer 2***	***37.94***	***265.55***	***66.39***

**Total**	**100.00**	**700.00**	**175.00**

**Table 4:** Pharmacotechnical properties of various ASAQ monolayer and bilayer tablet formulations

									30154C01	
									
	Batch n°	20454	30411	30412	30436	30437	30438	30439	layer AQ	layer AS
Formulation	tablet	monolayer							bilayer	
	
	granulation	wet aq.	dry							

Blend control	Flowability (s/100 g)	5	6	9	11	11	7	11	NA	NA
	
	Hausner index (Vo/Vf)	1.25	1.19	1.21	1.23	1.10	1.20	2.24	1.09	1.41
	
Tablet control	Mass (mg)	589.2	622.6	636.7	623.4	622.6	613.8	624	434.5	265.5
									
									700	
	
	Hardness (N)	61	80	63	78	68	74	71	46	
	
	Friability (%)	1.50	1.27	1.39	1.80	2.55	2.23	1.79	0.31	
	
	Residual humidity (%)	6	ND*	ND**	1.84	1.44	1.19	1.07	1.49	1.31
	
	Disintegration (min)	3.0	1.8	1.6	1.5	1.4	6.1	4.1	< 1.5	

Key: NA: data not available

*Residual humidity not determined, Karl Fischer water content 6.35%.

** Residual humidity not determined, Karl Fischer water content 6.33%.

Bilayer tablets showed excellent results in pre-stability studies (Table 5), as APIs content remained stable over three months at 40°C/75%RH and no DHA was detectable, contrarily to monolayer tablets or even to commercially available single-agent reference AS tablets, Arsumax^® ^(Sanofi-Aventis).

**Table 5:** AS and DHA recovery after 3 months in accelerated aging conditions (40°C/75%RH)

	AS content (%)	DHA content (%)
Arsumax^®^	> 95	3.3

Monolayer ASAQ tablet	97.8	3.4

Bilayer ASAQ tablet	99.1	< LOQ

Comparison between monolayer tablets with pH regulator (batch n° 30411), bilayer high dosage tablets (batch n° 40166) and commercially available reference of AS tablets, Arsumax^®^

**Figure 5:**
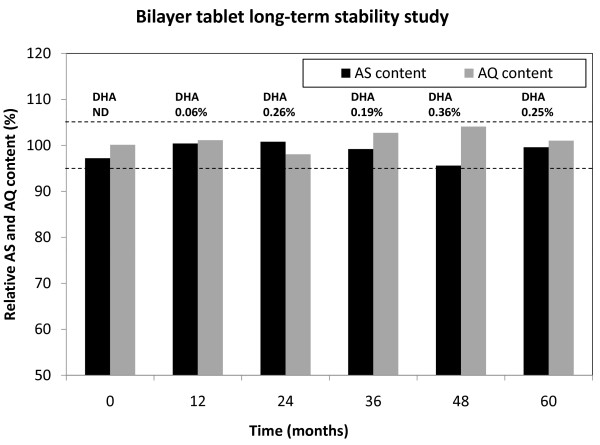
**Long-term stability study of ASAQ bilayer Aluminium blistered tablets in ambient conditions**. AS & AQ content (Y axis) and DHA content (cited above histogrammes) of batch n° 30154C04. Key: AS = artesunate; DHA = dihydroartesunate; AQ = amodiaquine; black dotted lines: specifications of AS&AQ content (100 ± 5%). Mean of 20.

Long-term and accelerated stability studies (60 months ambient conditions, see Figure 5; 12 months at 30°C/65%RH; and 6 months at 40°C/75%RH see Table 6) were then started.

**Table 6:** Drug recovery (%) in ASAQ bilayer tablets after 6 months in various aging conditions.

		25°C/60%RH	30°C/65%RH	40°C/75%RH
High dosage tablets	AS (%)	100.2	100.7	96.1
	
	AQ (%)	100.3	102.0	99.6

Low dosage tablets	AS (%)	102.0	102.0	99.2
	
	AQ (%)	101.7	102.7	101.8

Batches 30154C03 and 30154C04 for high and low dosage respectively, DHA < 0,5%

**Figure 6:**
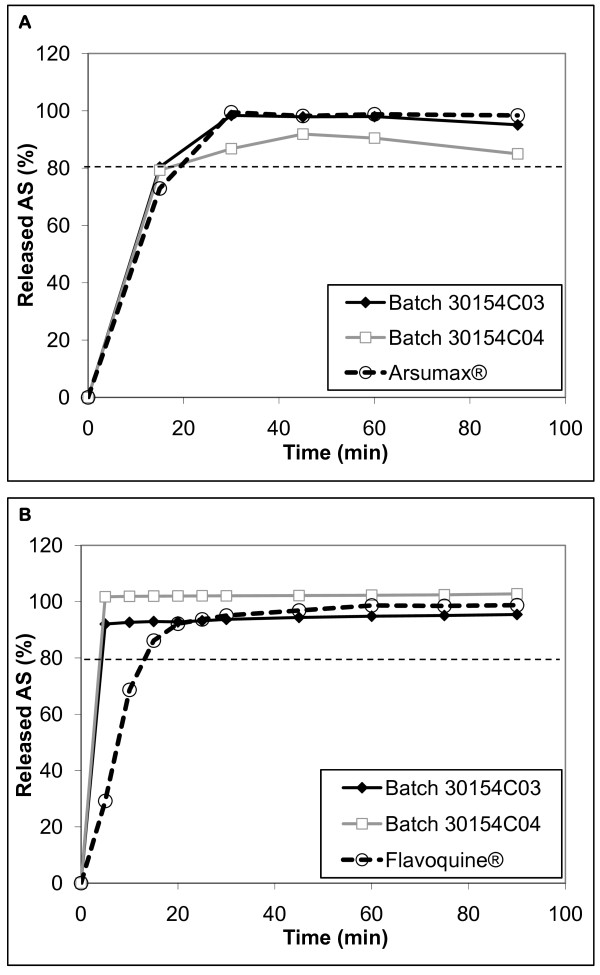
**Dissolution profiles of AS and AQ bilayer ASAQ tablets compared to commercially available reference tablets**. Key: Panel A: dissolution profiles of AS in high (30154C03) and low (30154C04) dosage bilayer ASAQ tablets compared to commercialized AS tablets Arsumax^®^ Panel B: dissolution profiles of AQ of high (30154C03) and low (30154C04) dosage bilayer ASAQ tablets compared to commercialized AQ tablets Flavoquine^®^.

Dissolution tests were performed on ASAQ bilayer tablets compared to the individually formulated standard product: AS Arsumax^® ^tablets and AQ Flavoquine^® ^tablets. Arsumax^® ^AS drug release profile was similar to bilayer AS drug release profile (Figure 6, panel A) whereas Flavoquine^® ^AQ profile was slightly delayed compared to the bilayer tablets (Figure 6, panel B). All profiles met Pharmacopoeia requirements for rapid drug release (i.e. > 75% drug released in 45 min).

## Discussion

### Pharmaceutical issues

AS is easily degraded by heat and aqueous conditions [9,10]. The key factors for AS degradation have been identified in pre-formulation studies as the combination of humidity (> 1%w/w) and temperature (≥40°C) on one hand (typically tropical conditions), and the presence of AQ (or another quinoline, e.g. quinine) and/or HCl on the other hand [2].

To prevent AS degradation several strategies were tried and many proved inadequate:

◦ Formulating AQ in the inner phase and AS in the external phase of tablets.

◦ Reducing residual water presence in the tablets either through wet or dry granulation. For alcoholic wet granulation, handling 95% ethanolic solution at industrial scale was not possible. Replacing wet granulation by dry granulation (AQ powder was not suitable for direct compression) was not sufficient to ensure AS stability and the addition of an hydrophobic agent (silicon dioxide) increased AS degradation, possibly through radical interaction [11].

◦ Manufacturing in controlled atmospheric conditions still led to > 5% AS tablet content loss after 3 months at 40°C/75%RH.

◦ Adding a pH regulator (CaCO_3_) improved partly AS stability but did not prevent degradation completely in dry granulation tablets even when combined with controlled atmospheric conditions manufacturing as some DHA was still detected.

◦ Controlled atmosphere conditions manufacturing did not meet cost requirements. Tablet protective coating was not considered as a suitable condition to avoid water contact.

In all the formulations above, residual humidity (see Table 4) in tablets exceeded the 1%w/w limit identified in pre-formulation studies for AS degradation [2]. Instead, experimental batches of bilayer ASAQ tablets, whereby the two active ingredients are physically isolated, met stability and dissolution criteria. Despite an initial water content slightly > 1%w/w (1.5% and 1.3% for AQ and AS granulate respectively) tablet drug content was stable for at least six months in accelerated stability conditions and for 60 months at 25°C/60%RH when packaged in alu/alu blisters (particularly adapted to storage in hot and humid areas like the tropics). In vitro drug release from these tablets was comparable to commercially available single-agent reference products. The disposition in healthy human subjects of the fixed-dose tablets was comparable, though not completely bio-equivalent, to the individually formulated drugs [12]. Furthermore, 60 months stability study of our pilot batch of ASAQ in ambient European conditions augurs well for the long-term stability of the formulation. If these results are confirmed in zone IV conditions, the product shelf life (currently 36 months) could be further extended, which would simplify drug procurement, storage and delivery.

### Project network

Establishing and running the operations of the FACT network proved challenging but rewarding. The project was funded through different mechanisms: following initial seed funds from WHO/TDR to the Biopharmacy Laboratory of Bordeaux2 University for validation of the pre-formulation [2], funding was provided by DND*i*, the European Commission INCO-DEV programme, MSFand TDR [13]. Most participating institutions donated personnel costs. When the project was passed onto Sanofi-Aventis for registration and commercialization in 2004, the company complemented with the chemistry, manufacturing and control part of the dossier for the product manufactured at industrial scale, which also included pharmaceutical and clinical studies. Overall, the project required five years from the bench to registration. Both the timelines and the costs are comparatively lower by orders of magnitude to the typical R&D process of a new chemical entity conducted by the pharmaceutical industry. In this case, the cost of failure in the early phases of pharmaceutical development was absorbed by the initial funders. Both, DND*i*, Sanofi-Aventis and TDR have continued to invest in phase 4 and implementation studies.

**Figure 7:**
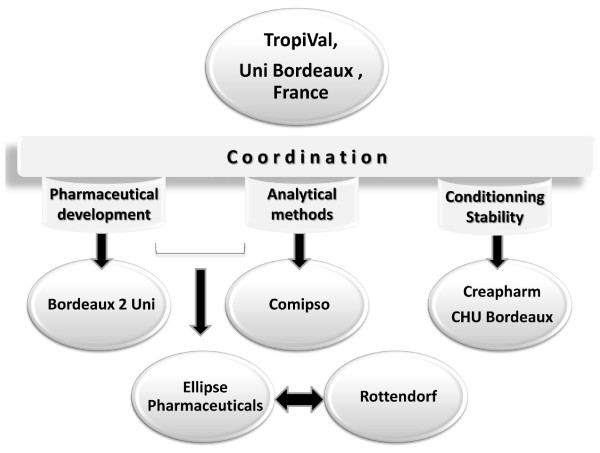
**Bordeaux team partner interaction**. Key: Uni = University

To conduct this project, the team in Bordeaux set up a public-private regional partnership, TropiVal, involving laboratories from Bordeaux 2 University for research on pre-formulations, Bordeaux Hospital for stability studies, the start-up COMIPSO for identification of degradation products, CREAPHARM (today UNITHER) for blistering, ELLIPSE Pharmaceuticals for process identification, development and validation of the bilayer ASAQ tablet, and ROTTENDORF Pharma for the manufacturing of the clinical batch (Figure 7). Each public partner accepted to work on the project either at cost or no fee (the Bordeaux University Hospital offering for many years free storage of ASAQ in regulatory conditions for stability studies), without counting human resources. Technology transfer from the University Sains Malaysia provided South-North links. However, the Bordeaux Tropival network was later terminated as it was found not sustainable; the university and the private partners could not secure the seed funds required to initiate projects ahead of larger partnerships with appropriate funding being established. Tropival's concept is now integrated within the *Pharmaceutical and analytical development applied to neglected diseases and counterfeit drugs *research unit of the University Bordeaux Segalen.

The product was developed as public good and together with DND*i*, ELLIPSE Pharmaceuticals decided to forsake the intellectual property rights associated to the process. The rights of producing and commercializing the product were initially transferred in 2005 by DND*i *to Sanofi-Aventis with a time-limited exclusivity agreement until registration. ASAQ (Coarsucam^® ^and Artesunate-Amodiaquine Whintrop^®^, Sanofi-Aventis) was first registered in 2007, three years after technology transfer, and WHO pre-qualified in 2009. The absence of intellectual property removed barriers to access and wider production as the exclusivity was time limited and is now expired. On the other hand, this approach may be difficult to replicate in all situations; while it relies heavily on partners absorbing part of the costs and risks, or working at cost, there is clearly no or limited prospects for return for those (typically in this case, the public sector) that allocate resources and take the risks inherent in the early phases of pharmaceutical development. Together, this paper and Kauss *et al *[2] provide a comprehensive account of the process that, through trials and errors, identified a suitable pharmaceutical form for industrial scale-up and was further developed for commercialization (Bompart F, Kiechel JR, Sebbag R, Pecoul B: Innovative public-private partnership to maximize the delivery of anti-malarial medicines: lessons learned from the ASAQ Winthrop experience, submitted). ASAQ is now registered and available in several malaria endemic countries.

## Conclusions

The development of a stable co-formulation containing AS and AQ in a bilayer tablet was a result of about three years of pharmaceutical development under the public-private partnership initiated by WHO and DND*i *with the not-for-profit and public sector in the lead. The development process was hampered by financial issues related to a lack of investment from the public sector. This lead initially to development goals focusing more on lowering the cost of manufacturing rather than on a more expensive optimization process based on the isolation of AS from AQ. This explains the delay in choosing an orientation towards a bilayer tablet. DND*i *has since established a strong fund-raising capacity building to allow completion of the FACT and other programmes. TropiVal is now part of a scientific team aiming to develop new pharmaceutical projects on drug development for neglected diseases. Moreover, this experience demonstrates that a network of scientists associated with industrial partners is able to lead to the production of improved tools for disease control. Although fundamental research is the initial aim of a public research institution, providing experience in applied research and technology transfer is likely to improve the relationship between academia and industry.

The resulting branded product (Artesunate-Amodiaquine Whintrop^®^) is currently made available at manufacturing cost to the national malaria control programmes of African and Asian endemic countries. As no intellectual property right was claimed by the network of scientists and industrials involved in the initial phase of the project, an exclusivity contract allowing technology transfer was signed between DND*i *and Sanofi-Aventis. The contract expired at the time of registration of ASAQ and the rights of producing and commercializing the product are now open and can be transferred to any other interested commercial partner.

## Abbreviations

ACT: artemisinin-based combination therapy; AQ: amodiaquine; (AQb: AQ base; AQH AQ: hydrochlorid salt); AS: artesunate; ASAQ: artesunate amodiaquine; CNRFP: National Centre for Research and Training in Malaria; DHA: dihydroartemisinin; DND*i*: Drugs for Neglected Disease initiative; FACT: Fixed artemisinin based combination treatment; FDC: fixed-dose co-formulations; MSF: Médecins Sans Frontières; PVP: polyvinylpyrrolidone; RH: relative humidity; TDR: Special Programme for Research and Training in Tropical Diseases of the WHO; WHO: World Health Organization.

## Competing interests

The authors declare that they have no competing interests.

## Authors' contributions

CL conceived the study, and participated in its design and coordination, managed the experiments at industrial scale, analysed and helped collecting the data, helped writing the draft manuscript and revised it critically. TK collected data, reanalysed and combined the data, wrote the draft paper, coordinated corrections, submission and response to reviewers of the manuscript. JRK conceived and designed the experiments, secured funding, helped analysing data and revised critically the manuscript. AC secured funding, contributed reagents/materials/analysis tools, and helped facilitate data collection. FF conceived and designed the experiments, carried out the experiments at laboratory scale, analysed and helped collecting the data. LT and SC carried out experiments at industrial scale for pharmacotechnical and analytical part respectively and analysed obtained data. LG conceived and designed the experiments, participated in data analysis and reviewed the manuscript. VN contributed first analytical methods and facilitated collecting data. BG participated to stability study experiments. KG analysed the data and revised critically the manuscript. NJW conceived and designed the experiments and participated in results discussion from a clinical standpoint. PLO conceived and designed the experiments, secured funding, analysed the data, helped writing the draft manuscript and revised it critically. PM conceived and designed the experiments, secured funding, analysed the data, wrote the draft manuscript and revised it critically before submission and reviewed it according to reviewers' comments. All authors read and approved the final manuscript.
